# Insights on cannabidiol's antiallodynic and anxiolytic mechanisms of action in a model of neuropathic pain

**DOI:** 10.1097/PR9.0000000000000774

**Published:** 2019-08-13

**Authors:** Marion Schott

**Affiliations:** EURIDOL Graduate School of Pain, University of Strasbourg, 67000 Strasbourg, France

## Abstract

Recent studies have shown that cannabidiol (CBD) could have a great therapeutic potential for treating disorders such as chronic pain and anxiety. In the target article, the authors propose that CBD modulates serotonergic transmission and reverses allodynia and anxiety-like behaviour in a rat model of neuropathic pain. Furthermore, this study shows an antinociceptive effect mediated by TRPV_1_ and partially by 5-HT_1A_ receptors, as well as an anxiolytic effect mediated by 5-HT_1A_ receptors.

**Commentary on:** De Gregorio D, McLaughlin RJ, Posa L, Ochoa-Sanchez R, Enns J, Lopez-Canul M, Aboud M, Maione S, Comai S, and Gobbi G. Cannabidiol modulates serotonergic transmission and reverses both allodynia and anxiety-like behavior in a model of neuropathic pain. PAIN 2019;160:36–150.

## 1. Introduction

Since 2018, one of the main internationally health-related debate concerns with the use of cannabis, also called marijuana. After the legalisation of cannabis in Canada in 2018, it is now the United States' turn to discuss this question. Nowadays, studies have highlighted many beneficial effects of cannabis, mainly due to cannabidiol (CBD), one of its most active components. Because it lacks addictive properties and euphoric effects, CBD is therefore an interesting potential pharmacological compound. Indeed, beneficial effects have already been found in a wide range of disorders such as chronic pain, anxiety, epilepsy, and psychosis.^2,12,13,14^ Cannabidiol is also interesting because it shows low affinity for the cannabinoid G-protein-coupled receptor CB_1_ but is an agonist of other receptors such as the transient receptor potential cation channel subfamily V member 1 (TRPV_1_) and the serotonergic 5-HT_1A_ receptor.^2,13^

Because pain is often in comorbidity with mood and anxiety disorders, more studies are now focusing on serotonin and serotonergic transmission, which is known to be implicated both in pain and in mood disorders.^7^ For instance, CBD has been shown to exert antidepressant and anxiolytic effects when injected in the dorsal periaqueductal gray and in the central nucleus of the amygdala.^1^ Despite these encouraging findings, few studies have analysed the effects of CBD treatment on serotonergic neurotransmission in the dorsal raphe nucleus (DRN), a brain region implicated in both mood disorders and pain.^10^

In this sense, we will discuss the study realised by De Gregorio et al.^3^ in which they reported the role of cannabidiol in the modulation of nociception, anxiety-like behaviour, and serotonergic transmission in a rat model of neuropathic pain.

## 2. Antiallodynic and anxiolytic mechanisms of action of cannabidiol

The first aim of this study was to determine CBD's mechanism of action on spontaneous firing activity of 5-HT neurons in the DRN. Cumulative doses of CBD (0.05–2 mg/kg intravenously [i.v.]) significantly decreased the 5-HT firing rate compared with vehicle injection (veh), and the dose of 0.25 mg/kg completely shut down neuronal activity. Pretreatment with the 5-HT_1A_ antagonist WAY100635 (0.3 mg/kg, i.v.) or the TRPV_1_ antagonist capsazepine (CPZ; 1 mg/kg, i.v.) both blocked the suppressive effect of CBD on 5-HT firing, which was not the case of the CB_1_ receptor antagonist AM251. These data suggest that CBD decreases 5-HT firing through 5-HT_1A_ and TRPV_1_ receptor-mediated mechanisms. Although the authors technically did not demonstrate that CBD induces TRPV_1_ desensitization, this is strongly supported by the study conducted by Iannotti et al.^9^ The authors, however, tested the extent to which repeated administration of CBD (7-day treatment, 5 mg/kg, subcutaneously [s.c.]) modulates 5-HT neurotransmission and found that this treatment increased 5-HT firing activity. Injections of cumulative doses of the 5-HT_1A_ receptor agonist LSD in rats pretreated with CBD or veh for 7 days showed that this increase of firing was likely due to a desensitization of the 5-HT_1A_ autoreceptors. Consequently, the authors analysed the effect of this increase of firing of the DRN 5-HT neurons in a model of neuropathic pain, the spared nerve injury (SNI) model.

Neuropathic pain was induced following Decosterd and Woolf's procedure.^4^ Rats were separated into 3 groups: SNI rats, naive rats that did not undergo any surgery, and sham rats that underwent a surgery that exposed the left sciatic nerve without further manipulation. Fifteen days after surgery, rats were treated with CBD for 7 days, from D0 to D7. Using von Frey filaments, rats were tested at baseline before the sham/SNI procedure and at day 15 (D0), 18 (D5), and 23 (D7). The data revealed that 7 days of CBD treatment significantly increased the paw withdrawal threshold in SNI rats at D7.

Immediately after the von Frey measurements, the same rats were tested in the forced swim test (FST) and open field test (OFT). Although the SNI rats showed both decreased time and number of entries in the center of the OFT compared with sham animals, 7 days of CBD administration restored these values. Spared nerve injury did not induce any significant changes in the immobility time in the FST. To further assess anxiety-like behaviour, a second cohort of rats underwent the elevated plus maze test (EPMT) and the novelty-suppressed feeding test (NSFT). The data reveal that cannabidiol increases the time spent in the open arms and decreases the latency to feed in a new environment for SNI rats, thus restoring levels found in sham animals.

After demonstrating the behavioural antinociceptive and anxiolytic effects of a repeated CBD treatment in SNI rats, the authors evaluated the role of TRPV_1_ and 5-HT_1A_ receptors in those effects. When treated with CBD + CPZ and tested with von Frey filaments, SNI rats showed a mechanical allodynia comparable to the SNI animals receiving vehicle. Interestingly, this treatment failed to block the anxiolytic effects of CBD in the OFT, EPMT, and NSFT because there were no significant differences between the SNI rats treated with CBD alone and those treated with CBD + CPZ. When treated with CBD + WAY, SNI rats exhibited mechanical thresholds that were significantly different from both SNI rats treated with veh and CBD alone, suggesting a partial involvement of 5-HT_1A_ receptors in CBD-mediated antinociception. Moreover, CBD + WAY treatment completely prevented the anxiolytic effects of CBD in the OFT, EPMT, and NSFT.

Then, the authors examined the effects of CBD treatment on the spontaneous activity of 5-HT DRN neurons in sham and SNI rats. The repeated administration of CBD led to a significant increase in the mean firing rate of DRN neurons in both sham and SNI rats. Cannabidiol also reduced the coefficient of variation and the number of neurons responsive to mechanical stimulation in SNI animals.

**Figure 1.**
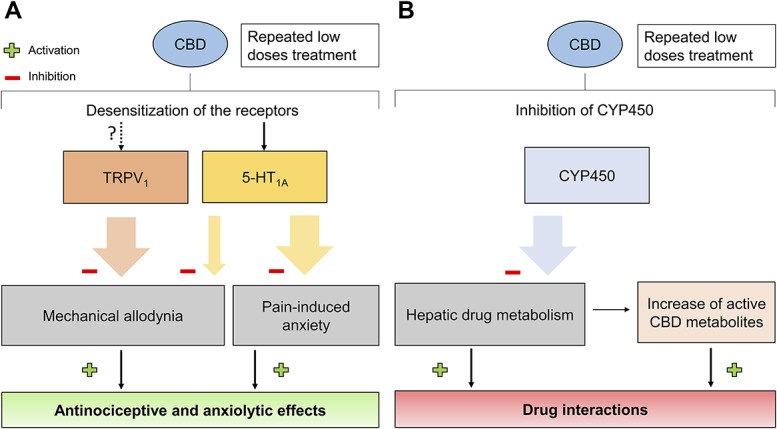
(A) Cannabidiol repeated treatment induces an antinociceptive and anxiolytic effect through action on TRPV_1_ and 5-HT_1A_ receptors. The “?” represents a mechanism of action that was not demonstrated in this specific study. (B) Although not mentioned in this study, CBD treatment could hypothetically lead to an increase in drug interactions and active drug metabolites when coadministered with other compounds. The large coloured arrows represent a complete effect, whereas the thinner coloured arrows represent a partial effect.

In summary, this study demonstrates the activity of CBD on DRN 5-HT neuronal activity, nociception, and anxiety-like behaviour through 5-HT_1A_ and TRPV_1_ receptors, without any direct involvement of CB_1_ receptors (Fig. 1A). Low-dose CBD treatment prevents mechanical allodynia through TRPV_1_ channels and partially through 5-HT_1A_ receptors. In addition, it reduces pain-induced anxiety-like behaviour through 5-HT_1A_ receptor-mediated mechanisms and prevents abnormal 5-HT neurotransmission in an SNI model.

## 3. Potential therapeutic uses and limitations

As previous work showed that a minimum of 20 mg/kg was necessary to exert antidepressant-like effects, the authors suggest the absence of effect of CBD in the FST to be due to the low dose used in the study (5 mg/kg). However, the rats may not show depressive-like behaviour because the FST was performed too early (23 days after surgery). This is supported by previous work indicating a depressive-like behaviour in SNI rats 7 weeks after the surgery.^6^ Altogether, the low CBD doses administrated having anxiolytic and antiallodynic effect prove that CBD-based compounds have a therapeutic potential to treat neuropathic pain and comorbid mood disorders. Indeed, the increase in the mean firing rate of DRN 5-HT neurons is a parameter observed in studies testing traditional anxiolytics and antidepressants.^5^ Although studies have shown that CBD intake only presents few nonsevere side effects,^8^ interaction between CBD and other drugs has not been thoroughly studied. This parameter is crucial because CBD oil is nowadays commercialised as a medication against chronic pain, Alzheimer disease, depression, stress, and anxiety. People taking this drug are probably suffering from one or several of these conditions and are likely to take other more traditional medication such as benzodiazepines against anxiety. However, previous work has shown that CBD impairs hepatic drug metabolism by inhibiting CYP4503A,^11^ an enzyme implicated in the metabolism of many drugs. If CYP450 is inhibited, some drugs administered will be less degraded by the enzyme, which could lead to an increase in active drug metabolites (Fig. 1B). This inhibition is potentiated by the route of administration, the dose, and the drug administered with CBD. Indeed, a recent phase I study showed that the administration of clobazam (benzodiazepine family) concomitantly with CBD led to a 47% to 73% increase of 7-OH-CBD, the active metabolite of cannabidiol.^11^

In conclusion, this study reported an interesting role of CBD in the modulation of serotonergic transmission and its antinociceptive and anxiolytic effects mediated by TRPV_1_ and 5-HT_1A_ receptors in a model of neuropathic pain. Cannabidiol could be a potential anxiolytic drug but further experiments on the interaction of CBD and other molecules should be conducted to be able to use CBD as medication.

## Disclosures

The author has no conflict of interest to declare.
